# Tocopherol Cyclases—Substrate Specificity and Phylogenetic Relations

**DOI:** 10.1371/journal.pone.0159629

**Published:** 2016-07-27

**Authors:** Jolanta Dłużewska, Renata Szymańska, Michal Gabruk, Peter B. Kós, Beatrycze Nowicka, Jerzy Kruk

**Affiliations:** 1 Department of Plant Physiology and Biochemistry, Faculty of Biochemistry, Biophysics and Biotechnology, Jagiellonian University, Gronostajowa 7, 30–387, Kraków, Poland; 2 Department of Medical Physics and Biophysics, Faculty of Physics and Applied Computer Science, AGH University of Science and Technology, Reymonta 19, 30–059, Kraków, Poland; 3 Institute of Plant Biology, Biological Research Centre of the Hungarian Academy of Sciences, Szeged, Hungary; 4 Department of Biotechnology, Faculty of Science and Informatics, University of Szeged, Szeged, Hungary; National Taiwan University, TAIWAN

## Abstract

In the present studies, we focused on substrate specificity of tocopherol cyclase, the key enzyme in the biosynthesis of the tocopherols and plastochromanol-8, the main plant lipid antioxidants, with special emphasis on the preference for tocopherols and plastochromanol-8 precursors, taking advantage of the recombinant enzyme originating from *Arabidopsis thaliana* and isolated plastoglobules, thylakoids and various model systems like micelles and thylakoids. Plastoglobules and triacylglycerol micelles were the most efficient reaction environment for the cyclase. In various investigated systems, synthesis of γ-tocopherol proceeded considerably faster than that of plastochromanol-8, probably mainly due to different localization of the corresponding substrates in the analyzed lipid structures. Moreover, our study was complemented by bioinformatics analysis of the phylogenetic relations of the cyclases and sequence motifs, crucial for the enzyme activity, were proposed. The analysis revealed also a group of tocopherol cyclase-like proteins in a number of heterotrophic bacterial species, with a conserved region common with photosynthetic organisms, that might be engaged in the catalytic activity of both groups of organisms.

## Introduction

Tocopherol cyclase is the key enzyme in the biosynthesis of tocopherols, the main plant lipid antioxidants, where it forms chromanol ring from hydroquinone precursors [1–3]. In recent years, it was found that this enzyme is also engaged in the synthesis of plastochromanol-8 (PC-8) from plastoquinol-9 (PQH_2_-9) in *Arabidopsis* [4–6]. Analysis of a variety of plant species [7] revealed widespread taxonomic distribution of PC-8 and it can be supposed that it occurs always together with tocopherols in photosynthetic organisms, although PC-8 is usually found at lower amounts than tocopherols [7]. Plastoglobules (PG) are major, if not the only chloroplast compartments where the enzyme is supposed to be localized [8–10].

Tocopherol cyclase was first isolated from the cyanobacterium *Anabaena variabilis* [11] and its substrate specificity was investigated [12]. Later, the enzyme was cloned from several organisms (*Synechocystis* sp. 6803, *Arabidopsis*, *Zea mays*) [13,14] and the recombinant proteins were further characterized in terms of substrate requirements and the enzyme kinetic parameters [14]. However, in none of these experiments, the ability of the cyclase to synthesize PC-8 was investigated. Moreover, some unresolved questions remained unanswered in these studies, regarding for example the lack of activity of the recombinant cyanobacterial enzyme [13] or the inactivity of the maize and *Arabidopsis* enzymes to form δ-tocopherol from its precursor [14].

In the present studies, we focused on the molecular factors affecting enzyme preference for tocopherols and PC-8 precursors, using recombinant enzyme from *Arabidopsis* and different experimental systems, like chloroplast fractions, liposomes and detergent micelles, containing natural and artificial enzyme substrates. Moreover, our study was complemented by bioinformatics analysis of the phylogenetic relations of the cyclases and sequence determinants of the enzyme activity.

## Materials and Methods

### Chemicals

All the chemicals were purchased from Sigma-Aldrich (St. Louis, MO, USA) unless otherwise indicated. Plastoquinone-2, ubiquinone-4 and menaquinone-4 were a kind gift of Hoffmann-La Roche (Basel, Switzerland). Dimethyl-phytylbenzoquinone and methyl-phytylbenzoquinone were synthesized by condensation of dimethylhydroquinone or methylhydroquinone, respectively, with natural phytol (Chem-Impex, Wood Dale, IL, USA) in diethyl ether in the presence of ZnCl_2_ and acetic acid. The obtained prenylquinols were oxidized with ferricyanide and purified by HPLC. Naphtoquinone-4 (gerenylgeraniol-naphtoquinone) was synthesized from naphtoquinone and gerenylgeraniol and purified as in the case of the phytol prenyllipids. Plastoquinone-9 (PQ-9) was purified as described by Kruk [15].

Triacylglycerols from linseed oil were purified by column chromatography on acidic aluminium oxide (grade I, Serva, Germany). One ml of the oil dissolved in 4 ml of hexane was applied on Al_2_O_3_ column (20 cm^3^), eluted with 100 ml of hexane, followed by 100 ml elution with 10% chloroform in hexane. The chloroform/hexane fraction was evaporated and used for further experiments.

Thylakoid lipids (monogalactosyldiacylglycerol, digalactosyldiacylglycerol, sulphoquinowozyldiacylglygerol and phosphatidyleglycerol) were purchased from Lipid Products (South Nutfield, Redhill, Surrey, U.K.) and used at the molar proportion of 50:25:12.5:12.5, respectively.

Trichloroacetic acid (TCA) esters of 8-hydroxyoctanoic acid (TC-C8) and of 12-hydroxydodecanoid acid (TC-C12) were synthesized from trichloroacetyl chloride and the corresponding hydroxyacids.

### Cyanobacteria and transformation

*Anabaena variabilis* 1403-4b and *Chlamydomonas reinhardtii* 11-32b were obtained from SAG collection (Göttingen, Germany). *Synechocystis* PCC6803 insertional mutant Δ*slr1737* and the vector to express tocopherol cyclase gene (SDX1) from maize were a kind gift of E.B. Cahoon.

The *Synechocystis* sp. PCC 6803 Δ*slr1737*/SXD1 transformant was prepared by conjugation. The plasmid pSynExp2MAIZE containing the SXD1 gene was introduced into *E*. *coli* DH5α harbouring pRK2013 helper plasmid (Biomedal, Cartujha, Spain) using 5 min transformation protocol [16]. The mutant *Synechocystis* was grown overnight to OD_720_ ~0.4. Five ml of *E*. *coli* containing both plasmids atOD_600_ ~ 0.25 was harvested by centrifugation, resuspended in 1 ml of BG11 medium and added to 48 ml of mutant *Synechocystis* culture and supplemented with 1 ml LB and 5 mM final concentration of glucose. This culture was kept overnight at 30°C in light in orbital shaker. The next day 250 μl of the culture was spread on a BG11 agar plate with no antibiotics in it and kept again overnight at 30°C in light. The cells were then transferred using a loop to a BG11 plate containing 25 μM spectinomycin and 10 μM carbenicillin. Selected colonies were repeatedly transferred to BG11 plates containing 25 μM spectinomycin and increasing concentration in four steps to final 50 μM carbenicillin. The transformant was grown on agar plates in BG11 medium in the presence of 50 μg/ml carbenicillin and 5 μg/ml chloramphenicol under low light (5 μmol photons/m^2^/s).

### Recombinant cyclase

Recombinant *Arabidopsis* tocopherol cyclase expressed in *Escherichia coli* was prepared using appropriate expression vector (a gift of R. Sadre) and purified according to the method described by Kumar et al. [14].

### Isolation of chloroplast fractions

Plastoglobules and thylakoids were isolated from 6-week-old *Arabidopsis vte1* mutant grown under 350 μmol photons/m^2^/s. Three rosettes were homogenized in 50 ml of HB buffer pH 8.4 (20 mM Tricine, 450 mM sorbitol, 10 mM EDTA, 10 mM NaHCO_3_, 5 mM sodium ascorbate, 1 mM MnCl_2_, 1 mM PMSF) for 30 s at full blender speed. The homogenate was filtered through nylon cloth and centrifuged at 5500*g* x 15 min. The chloroplast pellet was suspended in the buffer medium used for enzymatic assays (see below), sonicated for 1 min with 50% duty cycle on ice and centrifuged at 100000*g* x 100 min on Beckman L7 centrifuge. The plastoglobules layer formed on the top of the solution was dispersed in 0.7 ml of the supernatant, while the thylakoid pellet was suspended in 1 ml of the buffer.

### Enzymatic assay

Enzymatic assays were performed in 200 mM K_2_HPO_4_-KH_2_PO_4_ buffer (pH 7.0), 4 mM dithiothreitol, 75 mM sodium ascorbate and 50μM substrate, unless otherwise indicated. An aliquot of 2 mM stock solution of a prenylquinone in ethanol was treated with a small grain of NaBH_4_ to obtain the reduced substrate and injected into the reaction medium. The reaction was started by the addition of the recombinant enzyme (protein concentration in the stock solution was 1–1.5 mg/ml). Enzymatic assays were performed under anaerobic conditions, using oxygen trap composed of glucose oxidase, catalase and glucose [17] at 28°C for the indicated time with slow agitation.

### Prenyllipid analysis

Prenyllipids of cyanobacteria, *Phaeodactylum tricornutum*, green algae and maize leaves were analyzed as described previously [18].

In the case of HPLC analysis of the isolated PG and thylakoid fractions, 100 μl of the reaction mixture was extracted with 300 μl of ethyl acetate (vortexed for 60 s), centrifuged shortly, then 150 μl of the upper organic layer evaporated in a stream of nitrogen, dissolved in the HPLC solvent and analyzed using C18 RP column (Nucleosil 100, 25 x 4 mm, 5 μm, Teknokroma, Barcelona, Spain) in methanol/hexane (340/20, v/v), flow 1.5 ml/min and simultaneous absorption detection at 255 nm and fluorescence detection at 290/330 nm excitation/emission as described previously [19].

In experiments with detergents and liposomes, 100 μl of the reaction mixture was extracted with 100 μl of ethanol and 500 μl of hexane (vortexed for 60 s), centrifuged shortly, then 250 μl of the upper organic layer evaporated in a stream of nitrogen, dissolved in the HPLC solvent and analyzed as above. For the following substrates: PQH_2_-2, PQH_2_-4, ubiquinol-4, menaquinol-4, naphtoquinol-4 and phylloquinol, Acclaim C30 RP column (Thermo Scientific) was used.

### Fluorescence measurements

Fluorescence measurements were performed with Perkin Elmer LS50B and Varian Cary Bio spectrofluorometers using 2.5/5 nm or 5/5 nm excitation/emission slits, in 25 mM Hepes buffer (pH 6.5) or in organic solvents of spectroscopic grade. Excitation wavelength was 290 nm. Emission spectra were measured in the range 290–400 nm.

### Bioinformatics studies

The protein sequences of tocopherolcyclase were downloaded from NCBI database and aligned using Clustal Omega algorithm [20]. The phylogenic tree was constructed using the output data from Clustal Omega and Newicktops algorithm (Mobyle Pasteur: http://mobyle.pasteur.fr/).

### Statistical analysis

Standard error (SE) was calculated according to the formula:

$$SE=\sqrt{\frac{\sum {\left(x-X\right)}^{2}}{n(n-1)}}$$

where *x* is value of the measurement, *X* is arithmetic mean of *n* measurements.

## Results

### Taxonomic studies

As cyanobacteria were not investigated for the presence of PC-8, we analyzed under this respect several species, whose genomes were sequenced (Table 1). In none of the strains that are able to synthesize tocopherols, PC-8 was found. In the other strains (*Thermosynechococcus elongatus*, *Synechococcus elongatus* 6301 and 7942 strains), lacking cyclase genes in their genomes [18], neither α-tocopherol (α-Toc) nor PC-8 were found, as expected (Table 1). Since it is possible that the inability of cyanobacterial cyclase to synthesize PC-8 results from different aminoacid sequence from that of higher plants, we analyzed *Synechocystis* PCC6803 tocopherol cyclase insertional mutant (Δ*slr1737*) complemented with maize cyclase (SXD1). The mutant was not able to form neither α-Toc nor PC-8, while in the Δ*slr1737*/SXD1 transformant, only α-Toc could be detected (Table 1) under variety of growth conditions (light intensity) and age of the culture. This indicates that inability of cyanobacterial cyclase to synthesize PC-8 is not due to different sequence from that of higher plants and results from other reasons. In maize leaves, both old and young, the content of PC-8 is significant as compared to α-Toc or both redox forms of plastoquinone (Table 2). Next, we analyzed the level of PC-8 in representatives of other groups of photosynthetic organisms, i.e. in *Phaeodactylum tricornutum* (diatom) where no PC-8 was detected (Table 1) and green algae (*Chlamydomonas reinhardtii*, *Chlorella pyrenoidosa*) where only very low amounts of PC-8 were found (Table 2).

**Table 1:** Analysis of selected strains of cyanobacteria and *Phaeodactylum tricornutum* for the presence of α-Toc and PC-8.

Species/strain	α-Toc	PC-8
*Synechococcus* sp. PCC 7002*	+	‒
Phormidium laminosum*	+	‒
*Anabaena variabilis* SAG 1403-4b	+	‒
*Synechocystis* sp. PCC 6803	+	‒
*Synechocystis* sp. PCC 6803 Δ*slr1737*	‒	‒
*Synechocystis* sp. PCC 6803 Δ*slr1737*/SDX1	+	‒
*Thermosynechococcus elongatus* BP-1*	‒	‒
*Synechococcus elongatus* PCC 6301*	‒	‒
*Synechococcus elongatus* PCC 7942*	‒	‒
*Phaeodactylum tricornutum*	+	‒

*- the data were based on ref. 18.

**Table 2:** Content of prenyllipids in selected green algae and maize leaves.

Prenyllipid	*Chlamydomonas reinhardtii*	*Chlorella pyrenoidosa*	*Zea mays* (old leaf)	*Zea mays* (young leaf)
	mol/100 mol chl			
α-Toc	4.05 ± 0.55	1.12 ± 0.03	35.8 ± 2.0	4.0 ± 0.2
PC-8	0.14 ± 0.03	0.040 ± 0.001	2.35 ± 0.10	0.47 ± 0.13
PC-OH	0.05 ± 0.01	0	5.45 ± 0.25	0.48 ± 0.04
PQH_2_-9	2.23 ± 0.41	0	17.7 ± 1.0	2.4 ± 0.3
PQ-9	0.54 ± 0.17	2.86 ± 0.04	2.3 ± 0.2	1.10 ± 0.03
PQ_tot_	2.77 ± 0,50	2.86 ± 0.04	20.0 ± 0.8	3.5 ± 0.3
PC_tot_	0.19 ± 0.04	0.040 ± 0.001	7.72 ± 0.1	0.95 ± 0.10
PC_tot_/α-Toc	4.7%	3.6%	22%	24%
PC_tot_/PQ_tot_	6.8%	1.4%	39%	27%

The data are means ± SE (n = 3). PQ_tot_ = PQ-9 + PQH_2_-9, PC_tot_ = PC-8 + PC-OH.

### Substrate specificity studies

Subsequently, we took advantage of *Arabidopsis vte1* cyclase mutant, accumulating both dimethyl-phytyl-benzoquinol (DMBQH_2_) and PQH_2_-9 (Table 3) that are substrates for the cyclase, resulting in γ-Toc and PC-8 production, respectively. From the mutant plants, chloroplast fractions were isolated: PG and PG-free thylakoids. Both fractions contained similar levels of the substrates (Table 3) and were incubated with the recombinant cyclase derived from *Arabidopsis* (Table 4). The obtained results show that for PG, reaction proceeded efficiently for DMBQH_2_, while PC-8 was formed in this system with a very low yield. In thylakoids, the reaction with DMBQH_2_ was very slow and PC-8 formation was not observed at all.

**Table 3:** The content of tocopherol cyclase substrates in *vte1 Arabidopsis* mutant in leaves and chloroplast fractions used for the determination of tocopherol cyclase activity.

Leaves/chloroplast fraction	DMBQH_2_	PQH_2_-9	PQ-9
	**mol/100 mol chl**		
Leaves	22.7 ± 1.8	14.6 ± 0.6	1.8 ± 0.1
Thylakoids (PG-free)	4.3 ± 0.5	4.2 ± 0.3	1.0 ± 0.2
	**μM**		
PG fraction	21.3	14.6	1.12
Thylakoid fraction (PG-free)	203	195.2	48

The data are means ± SE (n = 3).

**Table 4:** Activity of recombinant tocopherol cyclase towards natural substrates in chloroplast fractions (plastoglobules and thylakoids) isolated from *Arabidopsis vte1* mutant.

Fraction/incubation time	γ-Toc/ DMBQH_2_	PC-8/ PQH_2_-9
PG/0h	0	0
PG/1h	65.5 ± 3.2	0.30 ± 0.08
PG/2h	81.2 ± 7.7	0.46 ± 0.05
PG/12h	105.5 ± 12.6	0.40 ± 0.12
Thylakoids/0h	0	0
Thylakoids/1h	1.04 ± 0.06	0
Thylakoids/2h	1.28 ± 0.09	0
Thylakoids/12h	1.18 ± 0.21	0

Activity is given in proportions (mol%) of the reaction products to the substrates after the indicated incubation time. For more details see Materials and Methods. The data are means ± SE (n = 3).

To investigate why there was such a difference in the reaction efficiency between PG and thylakoids, we analyzed the reaction rate in model systems where the substrates were incorporated into various detergent micelles or liposomes (Table 5). The reaction was most efficient in methyl-β-cyclodextrin (called cyclodextrin afterwards) solution for all the substrates, followed by detergent-free substrate micelles (substrates dispersed only in the buffer) and triacyglycerol micelles (TAG) that could be a structural model of PG. In all the micellar systems, γ-tocotrienol (γ-Tt) formation was slightly less efficient than that of γ-Toc, while PC-8 formation was not observed in any of the applied systems. Non-ionic detergents (DM, OG, Triton X-100 reduced) were totally inhibiting the reaction, while ionic sodium cholate was highly inhibitory. When the substrates were incorporated into liposomes, the reaction was observed for DMBQH_2_ only, but proceeded very slowly (Table 5). If we compare the reaction efficiency for DMBQH_2_ in TAG micelles and thylakoid lipid (TL) liposomes, that are model systems of PG and thylakoids, respectively, evidently the former system is more efficient in the reaction.

**Table 5:** Relative activity of recombinant tocopherol cyclase in different detergent and lipid systems.

Lipid system	γ-Toc (DMBQH_2_)	γ-Tt (PQH_2_-4)	δ-Toc (MBQH_2_)	PC-8 (PQH_2_-9)
0.5 mM cyclodextrin	100	87.3 ± 5.0	6.0 ± 1.9	0
Buffer	74.5 ± 5.1	63.2 ± 5.9	2.2 ± 0.8	0
0.5% DM	0	0	n.d.	0
1% OG	0	0	n.d.	0
0.025% Triton reduced	0	0	n.d.	0
0.5% cholate	10.2 ± 6.1	8.0 ± 4.1	n.d.	0
0.5 mM TAG	65.1 ± 4.2	45.4 ± 5.5	n.d.	0
EYL liposomes	~0	n.d.	n.d.	0
EYL liposomes/12h	2.6 ± 0.4	n.d.	n.d.	0
TL liposomes	~0	n.d.	n.d.	0
TL liposomes/12h	20.0 ± 4.8	n.d.	n.d.	0

Activity is expressed in terms of relative concentration of the reaction products (chromanols) in the reaction mixture after 30 min of incubation, unless otherwise indicated. Initial concentration of substrates was 50 μM. For more details see Materials and Methods. The data are means ± SE (n = 3). n.d.—not determined.

In the previous study [14], it was found that the recombinant cyclase was not active in methyl-phytyl-benzoquinol (MBQH_2_) to δ-Toc conversion. In our case, this reaction was slow but measurable (Table 5). We also tested, in the presence of cyclodextrin, other potential cyclase substrates such as PQH_2_-2, ubiquinol-4, menaquinol-4, naphtoquinol-4 and phylloquinol. However, none of the prenylquinols was active in the reaction.

### Fluorescence measurements

In order to investigate the reason why PQH_2_-9 is a poor substrate in the systems studied in contrast to DMBQH_2_, we took advantage of the intrinsic fluorescence of prenylquinols [21], which is sensitive to the medium polarity. Therefore, the possibly different membrane localization of hydroquinone rings of both substrates should be reflected in their different fluorescence yield. The data in Table 6 indicate that the fluorescence yield is the highest in solvents of the highest and medium polarity, especially in those containing hydroxyl group, while it is considerably lower in hydrophobic solvents, such as hexane or ethyl acetate. Fluorescence is completely quenched in chloroform, which is due to the presence of the heavy atom [22], and is also very low in dimethylformide because of the effect of the amine group [23,24]. The fluorescence efficiency of the prenylquinols in the systems where cyclase activity was measured (Table 7), is the highest in micelles of non-ionic detergents and the lowest in cyclodextrin, TAG and the buffer. The fluorescence yield of PQH_2_-9 is evidently lower than that of DMBQH_2_ in the buffer and TAG (Table 7). The fluorescence yield of all the prenylquinols increases with the decrease in their content in TL liposomes and at the lowest prenylquinol/membrane lipid proportions, the fluorescence intensity is the highest for PQH_2_-4, followed by PQH_2_-9 and DMBQH_2_.

**Table 6:** Fluorescence quantum yield (*ϕ*) of PQH_2_-4 in solvents of different polarity.

Dielectric constant (ε)	Solvent	Fluorescence quantum yield (*ϕ*)
37.5	acetonitrile	0.089
36.7	dimethylformamide	0.001
32.7	methanol	0.092
24.5	ethanol	0.117
17.5	*n*-butanol	0.094
10.3	*n*-octanol	0.082
7.6	tetrahydrofuran	0.055
6.0	ethyl acetate	0.017
4.8	chloroform	0
1.9	hexane	0.019

The data were calculated assuming *ϕ* in ethanol as 0.117 [21].

**Table 7:** Relative fluorescence intensity of the prenylquinols in different lipid systems.

Lipid system	Relative fluorescence intensity (I/I_methanol)_		
	PQH_2_-4	DMBQH_2_	PQH_2_-9
Buffer	0.024 ± 0.001	0.178 ± 0.003	0.053 ± 0.003
Buffer—10 μM prenyllipid	n.d.	0.156 ± 0.004	0.080 ± 0.002
0.5% DM	0.452 ± 0.015	0.494 ± 0.010	0.480 ± 0.009
1% OG	0.060 ± 0.002	0.317 ± 0.006	0.280 ± 0.005
0.025% Triton reduced	0.129 ± 0.004	0.251 ± 0.006	0.21 ± 0.01
0.5 mM cyclodextrin	0.044 ± 0.002	0.098 ± 0.002	0.081 ± 0.003
0.5% cholate	0.037 ± 0.002	0.062 ± 0.002	0.061 ± 0.002
0.5 mM TAG	0.051 ± 0.002	0.133 ± 0.003	0.073 ± 0.004
EYL liposomes -1:10	n.d.	0.190 ± 0.005	0.181 ± 0.008
TL liposomes– 1:10	0.133 ± 0.002	0.151 ± 0.003	0.158 ± 0.005
TL liposomes– 1:25	0.220 ± 0.005	0.167 ± 0.003	0.202 ± 0.003
TL liposomes– 1:50	0.292 ± 0.004	0.225 ± 0.005	0.270 ± 0.003
TL liposomes– 1:100	0.392 ± 0.001	0.29 ± 0.01	0.362 ± 0.005
TL liposomes– 1:200	0.57 ± 0.02	0.39 ± 0.02	0.50 ± 0.01

Concentration of the prenylquinols was 50 μM, unless otherwise indicated. In the case of liposomes, membrane lipid concentration (EYL or TL) was 0.5 mM and the numbers denote prenylquinol/lipid proportion (mol/mol). The data are means ± SE (n = 3). n.d.—not determined.

**Fig 1:**
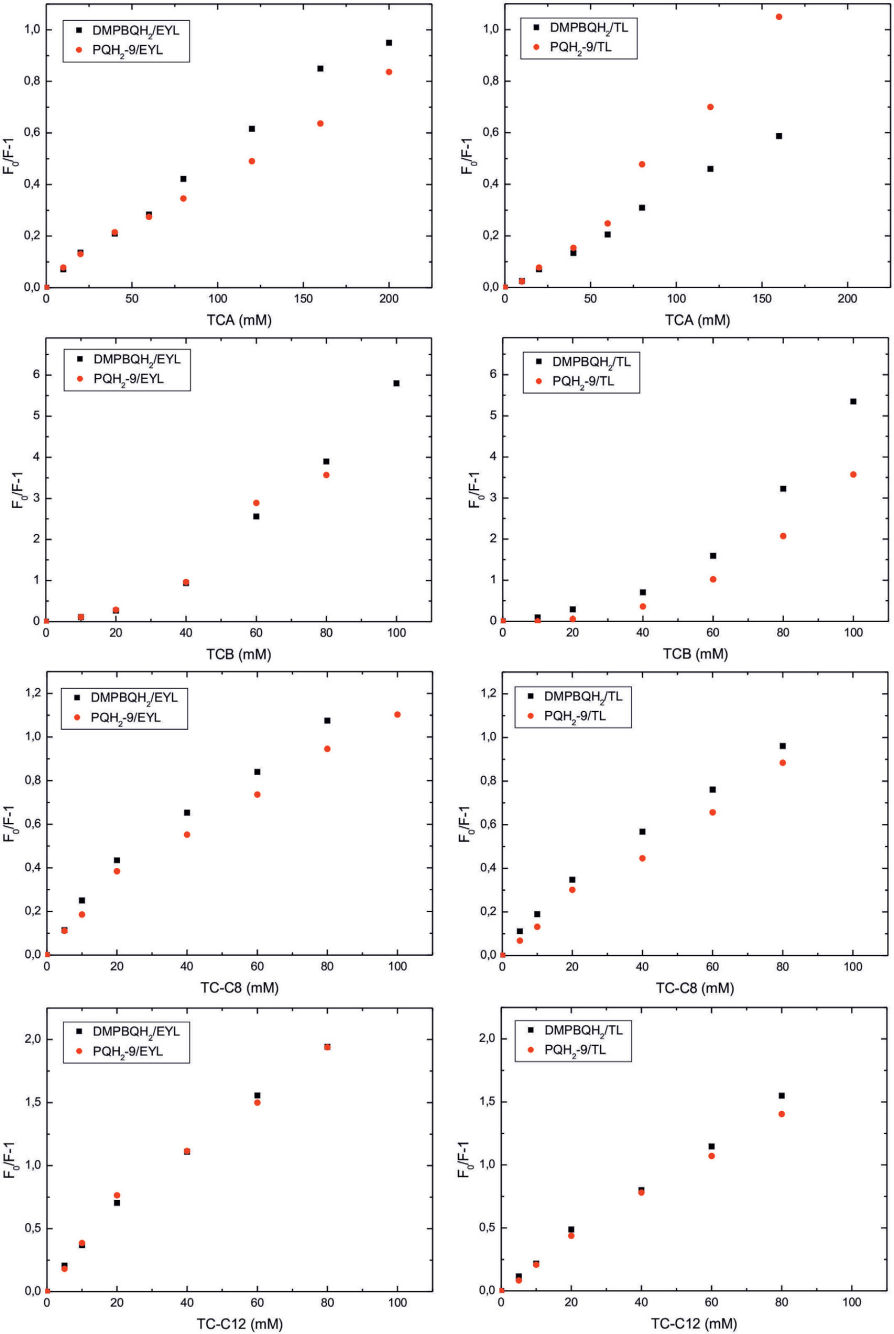
Stern-Volmer plots of prenylquinols fluorescence quenching. The quenching was performed in egg yolk (EYL) or thylakoid lipid (TL) liposomes by trochloroacetic acid (TCA), trichlorobutyric acid (TCB), TCA ester of 8-hydroxyoctanoic acid (TC-C8) and TCA ester of 12-hydroxydodecanoid acid (TC-C12) sodium salts. Prenyllipid/membrane lipid molar proportion was 1/10.

The other method used to determine the localization of fluorescent rings of the molecules in membranes is their fluorescence quenching by water or lipids soluble quenchers. For that purpose, we used water-soluble sodium salts of trichloro-fatty acids of different length as quenchers of prenylquinol fluorescence in egg yolk lecithin (EYL) and TL liposomes at a different depth in the membrane (Fig 1). For TCA, quenching of PQH_2_-9 in EYL liposomes was lower than that of DMBQH_2_, however in TL liposomes the effect was opposite. For TCB in TL liposomes and TC-C8 in both types of liposomes, the quenching of PQH_2_-9 was lower than for γ-Toc precursor, indicating that hydroquinone rings of PQH_2_-9 molecules are localized deeper in the liposome membranes.

### Bioinformatics analysis

**Fig 2:**
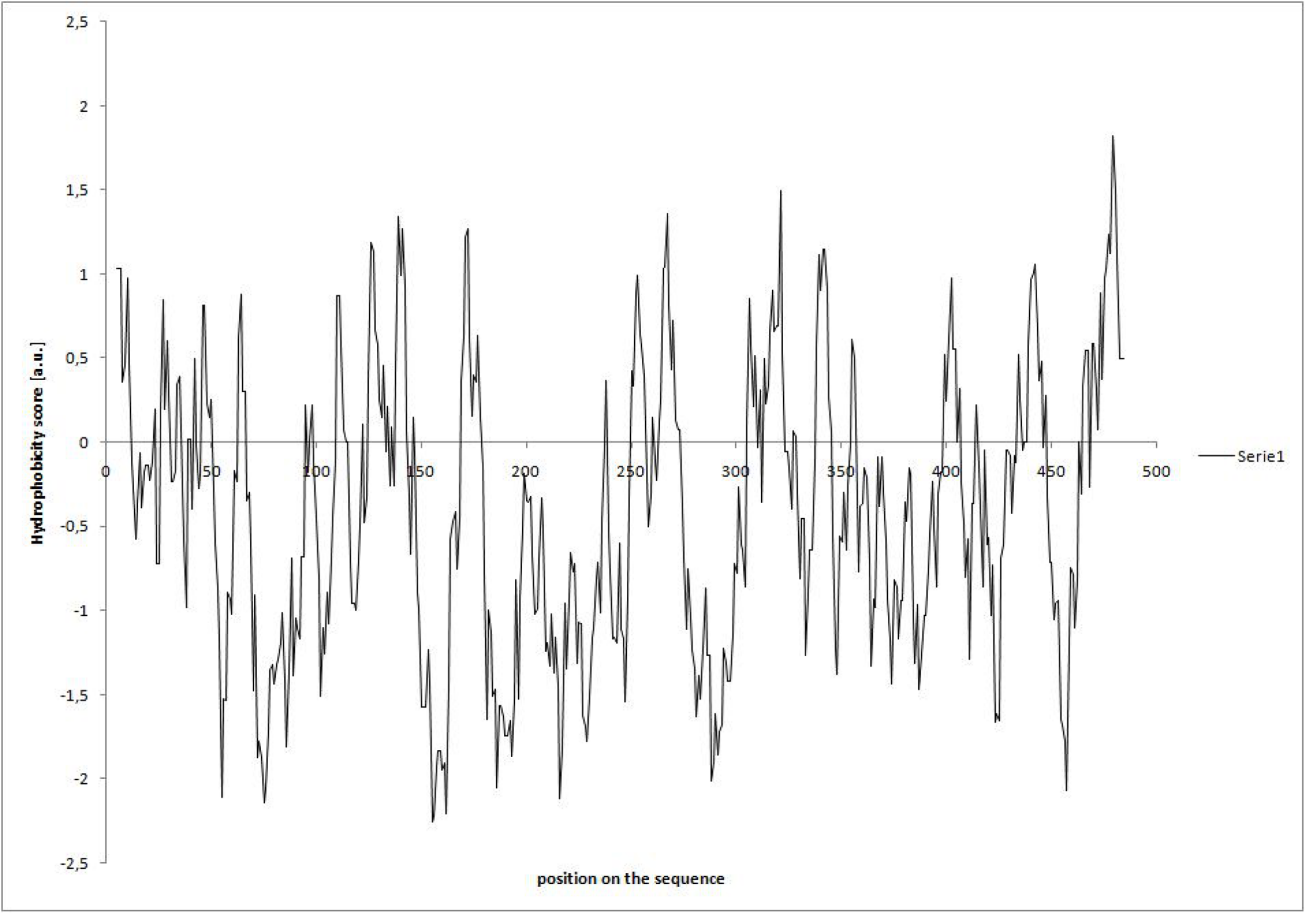
Hydrophobicity analysis of tocopherol cyclase aminoacid sequence from *A*. *thaliana*. Kyte and Doolittle hydrophobicity scale was used and widow size was set on 9. The analysis was performed using ProtScale server [25].

The literature data indicate that tocopherol cyclase is localised in PG [8–10] and our data show that in this chloroplast compartment, the recombinant cyclase is most active. To interact with the hydrophobic substrates (DMBQH_2_, PQH_2_-9), the enzyme must be attached to the PG surface and must be embedded to some extent within the PG structure. However, the hydophobicity profile of *Arabidopsis* cyclase (Fig 2) shows that there are no evident protein regions of increased hydrophobicity. Similar data were obtained for tocopherol cyclases from other species.

**Fig 3:**
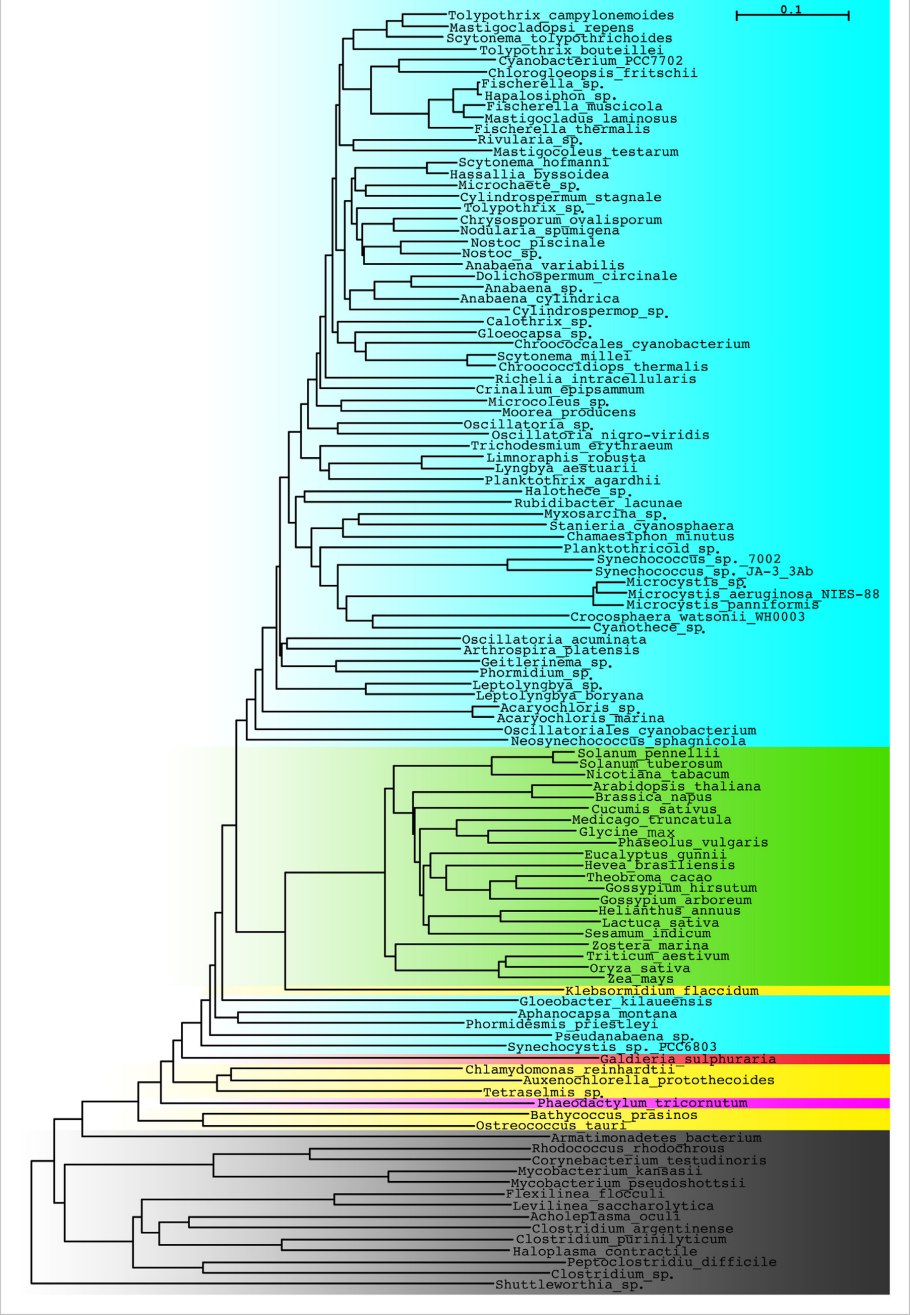
The unrooted phylogenetic tree of tocopherol cyclase sequences from different organisms. Bacterial proteins similar to tocopherol cyclase are included as an out-group. Blue- cyanobacteria, green—higher plants, yellow—green algae, red—red algae, pink—diatoms, gray—bacteria.

Presently, there are many tocopherol cyclase sequences available from species of different systematic groups (Fig 3), that are mainly based on whole genome sequences. The majority of cyanobacterial sequences form a separate branch on the phylogenetic tree, similarly as higher plants. The group of green algae is interlaced by the diatom *Phaeodactylum tricornutum* and is close to the red algae *Galdieria sulphuraria*. Among green algae, *Klebsormidium flaccidum* (*Charophyta*) is evidently the most closely related species to land plants that is in line with its systematic position based on whole genome sequence [26]. Interestingly, there is a group of non-photosynthetic bacteria having in their genomes cyclase-like protein (Fig 3).

It was previously found [13] that cyanobacterial cyclase sequences have considerably shorter C-terminal region of the protein than that of the higher plants and this region might be important for the enzyme activity. It cannot be also excluded that this difference is responsible for the enzyme specificity. The alignment of the protein C-terminal region of the available protein sequences (S1 Fig) shows that cyanobacterial sequences are evidently truncated as compared to those of other photosynthetic organisms. The length of the C-terminal region of the red algae protein is in between those of cyanobacteria and higher plants. The C-terminal regions of the green algae cyclases are of the same length as those of higher plants, while that of the diatom is even longer. Among higher plants, the C-terminal region of *Gossypium hirsutum* protein is exceptional, as it is considerably shorter than that of other higher plants. Taking into account that the length of C-terminal regions of *Chlamydomonas reinhardtii* and *Phaeodactylum tricornutum* proteins are similar to those of higher plants, it can be concluded that the extended C-terminal end is not important for tocopherols/PC-8 specificity of the cyclase.

**Fig 4:**
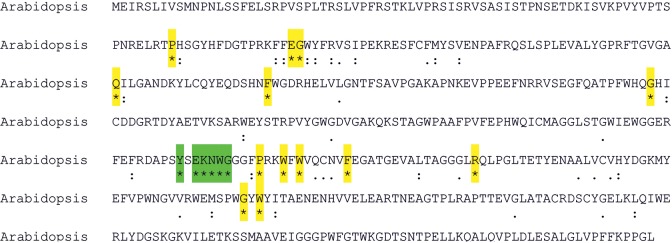
The conserved aminoacid residues of tocopherol cyclases from the photosynthetic organisms shown in Fig 3, marked on the sequence of *A*. *thaliana*. In the analysis, bacterial sequences were excluded. An asterisk indicates fully conserved residue. A colon marks residues with strongly similar properties. A period indicates residues with weakly similar properties. The analysis was performed using Clustal Omega algorithm. Fully conserved aminoacids were highlighted in yellow or green (the most conserved region probably engaged in the catalytic reaction).

Alignment of all the available sequences of cyclases of photosynthetic organisms, revealed 19 conserved aminoacid residues (Fig 4). Among them, the motif YxEKNWG is especially worth attention in light of the possible function of this region in the catalytic reaction of the enzyme.

Alignment of the 19 bacterial sequences of tocopherol cyclase-like proteins with that of *Arabidopsis* (S2 Fig) shows that the bacterial proteins have many identical/similar aminoacids to the higher plant sequence, ranging from 19/34 for *Peptoclostridium difficile* to 91/350 for *Rhodococcus rhodochrous* among 488 aminoacids in the *Arabidopsis* sequence. The most conserved region in the *A*. *thaliana* sequence (YxEKNWG) is clearly more or less preserved in many heterotrophic bacteria, being identical for *Haloplasma contractile*, *Clostridium purinilyticum* and *Corynebacterium testudinoris*. This indicates that this motif is probably also important for the activity of bacterial proteins of unknown function. Moreover, the neighbouring motif FP+xWxW+QxNxF is also frequently encountered.

## Discussion

Our study showed that none of the analyzed cyanobacterial species showed the presence of PC-8. Even the transformant of *Synechocystis* sp. PCC 6803, bearing tocopherol cyclase gene of maize instead of the cyanobacterial gene, was lacking PC-8 but it was able to synthesize α-Toc, as the wild type. PC-8 was also not detected in the diatom *Phaeodactylum tricornutum* and it occurred at very low levels in the green algae investigated. These data indicate that differences in the protein sequence among the investigated organisms are not critical for the enzyme substrate specificity, as far as synthesis of tocopherols and PC-8 is concerned. Localization of tocopherol cyclase in PG of higher plants and considerably higher activity of the cyclase reaction in the isolated PG fraction than in isolated thylakoids (Table 4) suggests that the efficient enzyme action and PC-8 synthesis is only possible in plastoglobules. Cyanobacteria and green algae were considered not to have PG, which might explain lack or low levels of PC-8 in these organisms. However, recent literature data indicate that PG are present in cyanobacteria, chloroplasts of green algae and other algae groups, although PG in these organisms are less abundant and are formed mainly under stress conditions [27–30].

Early studies on substrate specificity of tocopherol cyclase from *Anabaena variabilis* [12] revealed that the enzyme converts substrates with the free hydroxyl group in the hydroquinone ring, substrates with a different number of methyl groups in the ring and having both saturated and unsaturated side chains. Therefore, the enzyme is also capable of forming tocotrienols [31] and tocomonoenols [32]. Among plastoquinol substrates with a side chain of different number of isoprenoid units (2–4), the highest activity was observed for the substrate with 4 units (83%), followed by that with 3 units (14%), while plastoquinol-2 was not active in this respect [12]. This indicates that the substrate side chain must have an appropriate hydrophobicity. On the other hand, in the recent studies [14] it was shown that in contrast to DMBQH_2_, MBQH_2_, which is the precursor of δ-Toc, was not active in the reaction. In our studies, both DMBQH_2_ and PQH_2_-4, differing only in the unsaturation of the side chain, showed similar activity in model systems (Table 5). Interestingly, MBQH_2_ was also converted by the recombinant enzyme to δ-Toc, but with the considerably lower yield. Among other analyzed substrates with the same side chain as that of PQH_2_-4 (ubiquinol-4, menaquinol-4, naphtoquinol-4 and phylloquinol) none of these were active. This indicates that the type of hydroquinone ring substituents is important for the activity. Analysis of ubiquinol as the potential substrate is important in light of the literature data on the occurrence and potential function of ubichromenol in plants, mammals and some microorganisms [33–36]. However, it cannot be excluded that this compound was an isolation artefact or it is formed non-enzymatically in these organisms. Unfortunately, in the previous studies [12,13] PQH_2_-9 was not studied as a potential substrate. In most of our systems, it was not active as PC-8 precursor and only minor amounts of PC-8 were formed in the isolated PG fraction. Probably PQH_2_-9 is considerably less active than the short-chain substrates, because of a different accessibility of the hydroquinone rings of these homologues to the enzyme. In the case of PQH_2_-9, its rings are probably buried deeper within the lipid structures studied.

Among the investigated model systems (Table 5), the highest enzyme activity was found when the substrates were dispersed in cyclodextrin, the buffer and TAG, where high aggregation of the substrate molecules occurs and hydroquinone rings of their molecules are buried within micelles to some extent. This can be concluded form the lowest relative fluorescence of substrate molecules in these systems (Table 7). If only low fluorescence was due to location of the substrates in the hydrophobic environment approximated by hexane, then their relative fluorescence should be not less than 0.2 (0.017/0.092, Table 6). The lower than that relative fluorescence of the prenylquinols in the investigated systems indicates their aggregation, as aggregation in known to quench fluorescence of prenylquinols [21]. In the case of PQH_2_-9, aggregation in the discussed systems is more pronounced. The dependence of prenylquinols fluorescence efficiency in liposomes on their content suggests that the lower their concentration in the membranes, the less aggregated molecules are, and relatively more molecules are localized close to the membrane surface. Similar conclusions were inferred previously from monolayer studies [37]. Deviation from linearity of Stern-Volmer plots in fluorescence quenching experiments (Fig 1), indicates heterogeneity of prenylquinols distribution in liposome membranes and in the case of TCB also mixed, dynamic-static character of quenching [38].

As it was suggested that tocopherol cyclase is a hydrophobic protein [12,13] we have analyzed the hydrophobicity profile of the protein but none of its regions showed increased hydrophobicity (Fig 2). A good water-solubility of the protein during isolation procedure of the recombinant protein and lack of tendency to form aggregates even at high concentration also indicates that cyclase shows no hydrophobic regions. Nevertheless, it must have some tendency to bind to the PG surface and to be embedded partially within its structure to interact with the hydrophobic substrates. It is possible that in vivo contribution of some other PG proteins facilitates such a penetration and certain post-translational modifications could be also important in this respect. It was found that tocopherol cyclase in PG is phosphorylated by ABC1K1/K3 kinases [10] and it has also potential myristoylation sites [13]. The latter modification could increase local hydrophobicity of the enzyme molecule and its penetration into PG droplets and enhance its affinity towards PQH_2_-9.

Tocopherol cyclases are unique proteins with no other known related proteins. The analysis of the available genomes indicates that only one gene of the cyclase is present in a genome, therefore no cyclase isoforms can be expected, however different splicing and posttranslational modifications can give rise to heterogeneity of the enzyme. Tocopherol cyclase (EC 5.5.1.24) belongs to a group of intramolecular lyases comprising several other enzymes forming rings of different terpenoids, however none of these enzymes shares an evident sequence homology with the cyclase.

Sequence alignment of the tocopherol cyclases from photosynthetic organisms (Fig 4) revealed several conserved aminoacid residues and especially the most conserved region of five neighbouring aminoacids could be important for the enzyme activity and catalytic properties. In the suggested enzymatic reaction mechanism [39] a proton donor aminoacid was proposed and one of the residues of the conserved motif could be engaged in this reaction.

One of the most unexpected findings of the phylogenetic analysis was identification of tocopherol cyclase-like proteins coded in genomes of non-photosynthetic bacteria, that contain more or less conserved catalytic motif identified in photosynthetic organisms. The possible function of these proteins deserves further study.

## Supporting Information

S1 Fig Alignment of C-terminal region of tocopherol cyclase sequences from the photosynthetic organisms and sequences of tocopherol cyclase-like proteins from bacteria indicated in Fig 3. The analysis was performed using Blast algorithm.
(PDF)

S2 Fig The alignment of tocopherol cyclase sequence from *A*. *thaliana* and sequences of bacterial tocopherol cyclase -like proteins. The numbers following the species name, indicate the number of identical/similar residues between the sequences being compared. In the middle row, an aminoacid symbol indicates fully conserved residue, while '+' indicates a residue with strongly similar properties. The most conserved region in the sequence of photosynthetic organisms, shown in Fig 4, is highlighted in green. The analysis was performed using Blast algorithm.
(PDF)
