# Supplementary material for: Tocopherol Cyclases—Substrate Specificity and Phylogenetic Relations
Source: PLoS One. 2016 Jul 27;11(7):e0159629. doi: 10.1371/journal.pone.0159629 (PMC4963081; doi:10.1371/journal.pone.0159629)
Supplement: S1 Fig — The analysis was performed using Blast algorithm. (PDF) [file pone.0159629.s001.pdf]

Chamaesiphon_minutus[WP_041549967.1]	---DRII---EAN--------SHNAGLEVGGA---GWDQAWAK--
Myxosarcina_sp.[WP_036485872.1]	---KTIV---MAN--------SNLAGLEIGGS---SWDEPWIVNA--
Stanieria_cyanosphaera[WP_015194825.1]	---KTIV---KAS--------TEVAGLEVGGF---PWNQAWKVVTE---
Microcystis_panniformis[AKV65950.1]	---KTII---NAS--------SGLAGLEIGG----IWPSAWIKR---
Microcystis_sp.[WP_008199571.1]	---KTII---DAS--------SGLAGLEIGG----IWPSAWIKQ---
Microcystis_aeruginosa[WP_002796597.1]	---KTLI---NSS--------SGLAGLEIGG----IWPSAWIKHL--
Oscillatoriales_cyanobacterium[EKQ69809.1]	--DRLIL---EAC--------SSLAGLETGG----SWDGVWRSV---
Neosynechococcu_sphagnicola[WP_036534715.1]	--DQLIL---AAT--------SDLGGLEVGGA---PWDTPWQV--
Synechococcus_sp._7002[WP_012306299.1]	---TPLI---QAT--------SDLGGLEVGGQ---WWRRDFG---
Crocosphaera_watsonii[CCQ53308.1]	---KILL---KAN--------SCLGGLEIGGS---PWDDSWIYG---
Planktothricoid_sp.[WP_054467593.1]	PNSPPIL---QAN--------SSLAGLETGGE---PWQETWRSPVSAVSRQQ-EAIGY-------------------------------------
Acaryochloris_marina[WP_049783828.1]	QGTTLLL---KAH--------SHLCGLEVGGG---PWQTAWIAEEAMSPPLDAAPWGLAAT----------------------------------
Acaryochloris_sp.[WP_050857422.1]	QGTTLLL---KAH--------SHLCGLEVGGG---PWQTAWIAEEAMSQPLDAAPWGLAAT----------------------------------
Rubidibacter_lacunae[ERN42735.1]	---RTRL---TAK--------SDTCGLEVGGI---PWPGPWSDRHPQPTSA-RRDCG--------------------------------------
Leptolyngbya_boryana[BAS58789.1]	---RLIL---EAT--------SSLAGLETGGS---PWDETWASGCGQVF--
Halothece_sp.[WP_015227537.1]	---NLIC---QAS--------STNCGLEVGGI---PWSSPWINE---
Phormidium_sp.[KPQ39197.1]	GEYQTIL---EAT--------SSQCGLEVGGG---PWNETWVVG---
Cyanothece_sp.[WP_012628024.1]	WGLALIV---KAQ--------STLCGLETGGG---PWEQVWTA--
Arthrospira_platensis[KDR56569.1]	--RRLIL---EAH--------SSLCGLETGGG---PWDETWVH--
Geitlerinema_sp.[WP_015172570.1]	HGWTPIV---EAS--------SSLGGLEVGGG---PWDEPWVSGR--
Oscillatoria_sp.[CBN58299.1]	GNSKIIL---EAQ--------SNLCGLETGGG---PWDEAWRSQSEGLAINGHFNC---------------------------------------
Oscillatoria_nigro-viridis[WP_015178838.1]	QRSRIIL---EAQ--------SSLSGLEVGGG---PWDEVWRSH---
Microcoleus_sp.[AFZ17727.1]	GKSKIIL---NAS--------SSVCGLEIGGG---PWQDAWVSSAKFPWVIYR--
Moorea_producens[WP_008181943.1]	DNVEPIL---IAE--------SKLCGLEVGGD---SWEKPWNSSAKLPWVL--
Oscillatoria_acuminata[WP_044197505.1]	NPGEIIF---QAT--------SSLCGVETGGG---PWTETWRSA---
Planktothrix_agardhii[KEI67078.1]	--NSIIL---EAT--------SSLGGVEVGGD---LWQETWKK--
Lyngbya_aestuarii[WP_023068270.1]	--KEIIL---EAK--------SDQCGVEVGGH---PWTEVWVN--
Limnoraphis_robusta[WP_046280920.1]	--NQVIL---FAK--------SDVCGVEVGGH---PWTDTWVN--
Trichodesmium_erythraeum[WP_047165208.1]	SHSKLIL---EAR--------SDLCGLEVGGG---SWEQAWVK--
Scytonema_millei[KIF18394.1]	GK-KLIL---EAK--------SSVCGLEIGGS---PWDSTWQK--
Chroococcidiops_thermalis[WP_015153712.1]	GK-KSIL---EAT--------SSVCGLEIGGN---PWDSAWQQ--
Chroococcales_cyanobacterium[WP_045053506.1]	GKSNVIL---SAH--------SSLCGLETGGS---NWEGAWLSS---
Crinalium_epipsammum[WP_041226015.1]	GKSKLIL---EAH--------SSLCGLEVGGG---SWEESWRSS---
Richelia_intracellularis[CDN10345.1]	TQQKIIL---KAQ--------SFLCGLEIGGG---SWENSWQSG---
Mastigocoleus_testarum[KST64378.1]	KKIKTIL---TAR--------SSLCGLEIGGG---SWNDPWSSSS--
Gloeocapsa_sp.[WP_015189813.1]	--QQSII---TAY--------SSLCGLETGGG---SWGTTWYSN---
Fischerella_thermalis[WP_016871339.1]	NQQEIIL---KAH--------SSACGLEIGGG---PWNNAWQSH---
Fischerella_muscicola[WP_016868113.1]	NHQEIIL---KAH--------SSACGLEIGGG---PWNDAWQSH---
Hapalosiphon_sp.[WP_053454991.1]	NHQEIIL---KAD--------SSACGLEIGGG---PWNDAWQSH---
Fischerella_sp.[WP_017310725.1]	NHTEIIL---KAD--------SSACGLEIGGG---PWNDAWQSH---
Mastigocladus_laminosus[KIY13639.1]	NHTEIIL---KAD--------SSACGLEIGGG---PWNDAWQSH---
Chlorogloeopsis_fritschii[WP_016874874.1]	SHRRTIL---KAR--------SSLCGLEIGGS---PWSDRWVSD---
Cyanobacterium_PCC7702[WP_017322824.1]	TERKTIL---KAH--------SCLCGLEIGGS---PWERSWQSH---
Tolypothrix_bouteillei[KIE07857.1]	EKGKTIL---RAS--------TFLCGLEIGGR---GWENSWQSR---
Scytonema_tolypothrichoides[WP_048867363.1]	GKRKTIL---KAR--------SFLCGLEIGGG---SWDNSWQSR---
Mastigocladopsi_repens[WP_017315776.1]	GKRKIIL---KAR--------TFLCGLEIGGE---PWDNSWQSH---
Tolypothrix_campylonemoides[KIJ76814.1]	GKRKTIL---KAR--------TFLCGLEIGGG---SWDNSWQSR---
Cylindrospermop_sp.[WP_057179079.1]	NHSTVIV---KAS--------SDVCGLEVGGD---SWENSWQFQ---
Calothrix_sp.[AFZ03630.1]	GKIKTIL---TAH--------SDLCGLEVGGE---NWNNSWQGG---
Rivularia_sp.[WP_015116287.1]	VESKTIL---KAT--------SDLCGLETGGG---PWDSSWLND---
Dolichospermum_circinale[WP_028082234.1]	SKSQVIL---NAS--------SNLCGLEIGGH---DWDNYWQSQ---
Anabaena_cylindrica[WP_015217110.1]	GKSKIIL---KAQ--------SHLCGLEIGGD---GWENIWQSL---
Anabaena_sp.[WP_016950282.1]	SKSKIIL---KAQ--------SHLCGLEVGGA---TWENIWQSS---
Anabaena_variabilis[ABA22348.1]	KNPLVIL---KAH--------SYLCGLEIGGD---SWSNSWQSS---
Nostoc_sp.[WP_044520539.1]	KNPLVIL---KAH--------SYLCGLEIGGD---SWSNSWQSS---
Cylindrospermum_stagnale[AFZ27795.1]	GKSKTIL---KAH--------SYLCGLEIGGG---TWNNSWQSP---
Microchaete_sp.[WP_017652428.1]	GKSQIIL---KAE--------SNLCGLEVGGG---AWDNSWHSR---
Hassallia_byssoidea[KIF35717.1]	TKSKTIL---KAH--------SFLCGLEVGGG---SWDNSWQSS---
Scytonema_hofmanni[WP_029634470]	TKSKTIL---KAH--------SFLCGLEVGGG---SWDNSWQSS---

Tolypothrix_sp.[WP_045872519.1]	GKPRIIL---KAQ--------SSLCGLETGGG---SWDDSWQSS---
Nostoc_piscinale[ALF55534.1]	KKSQSIL---KSQ--------SSLCGLEIGGG---SWDSSWHSS---
Chrysosporum_ovalisporum[CEJ45205.1]	KKSTTIL---KAQ--------SSLCGLEIGGG---SWDNSWQSS---
Nodularia_spumigena[AHJ29258.1]	GKSSTIL---KAE--------SSLCGLEIGGG---SWDNSWHSK---
Pseudanabaena_sp.[AFY69362.1]	---GLQL---RAS--------DNQAALEIGGD---PWPEPWQFKSAQI---
Synechococcus_sp._JA-3_3Ab[WP_011429551.1]	-VWTQVL---AAS--------SSQAGLEVGGE---V-PRSWPGHC--
Synechocystis_sp._PCC6803[BAA17775.1]	-GHGLIV---QGE--------TDTAGLEVGGD----WGLTEENLSKKT-VPF---
Gloeobacter_kilaueensis[WP_023172101.1]	GTRAVLF---EGS--------TRLGGLETGGG---PWEGEWQFRC--
Aphanocapsa_montana[KIF13693.1]	SDQSLIL---KAE--------THLAALEVGGK---GWQQPWHFVQG-A-I---
Leptolyngbya_sp.[ESA32374.1]	-SHQPIL---RAH--------SKQAGLEVGGE---DWHTLWQHSS--
Phormidesmis_priestleyi[KPQ33913.1]	GTDRLIV---ETT--------SQLAGLEVGGG---PWRDRWSNR---
Galdieria_sulphuraria[EME26452.1]	-TGCRVL---DDY--------SNLAAVEVGGQDKDCWKHVWNDNVLQLPLFLRNAIQYF-DGTE-------------------------------
Bathycoccus_prasinos[CCO14153.1]	-KPIFEN---ASS--------GSTACLEVGGG---PWEAPWEIQAD-VKDPLAGALAMTDVNVELVQSVGK-FFGLDVIPGL-------------
Ostreococcus_tauri[CEF96598.1]	-VETIID---AAM--------SSTACLEIGGG---PWVSPYRAVAE-IRTPLGPLMGLP-VDVNAVAKILS-PL-GDLLPGL-------------
Klebsormidium_flaccidum[GAQ89951.1]	MRGKVIV---HAT--------SSMCALEVGGG---PWWQTWKKTAR-YEEPVRSLMDLP-L--GNVLQAIPPNL---QPPGL-------------
Chlamydomonas_reinhardtii[EDP09189.1]	KQGQPLV---DAT--------SSTAALEVGGG---PWWSAWRARAE-MKEPFRSLVTLP-LDVAGLGQLVPEPL---RPPGL-------------
Auxenochlorella_protothecoides[KFM23407.1]	APPVVDC---VSL--------EETGAVEVGGG---PWWSGWKARAA-MAGGVKALLNLP-VDVEGLTGWLPGGL---RPKGL-------------
Tetraselmis_sp.[JAC69290.1]	RHPVPFI---DAT--------SSTAALEVGGG---PWWSAWKAKAR-MAEPLRTLVSMP-VE--AVVNRLPQPI---RPPGL-------------
Phaeodactylum_tricornutum[EEC42647.1]	PGPPLID---RAT--------SSQGGAEIGGG---PWWSTWEATSV-VKQPLKALLQIP-ARVQNYRRKRSQA-----------WSAMSTEENSK
Zea_mays[AFW70910.1]	SKGEMIL---DAT--------SNMAALEVGGG---PWFNGWKGTTV-VNEVVNNIVGTP-VDVESLLPI-P-FL---KPPGL-------------
Oryza_sativa[BAS78094.1]	GKGKMIL---DAT--------SNMAALEVGGG---PWFNGWKGTTV-SNEIVNNVVGTQ-VDVESLFPI-P-FL---KPPGL-------------
Triticum_aestivum[ABE41800.1]	SKGKIIL---DTT--------SNMAALEVGGG---PWFNGWKGTTA-TNEPVNNIVGTQ-IDVESLFPI-P-FL---KPPGL-------------
Zostera_marina[KMZ57637.1]	SKGKMIL---DVT--------SNMAAVEVGGG---PWYSTWKGTTS-SPRLLSEALKVP-IDLESVLSV-P-FL---KPPGL-------------
Sesamum_indicum[ABW98674.1]	SEGKVIL---DVT--------SNMAAVEVGGG---PWFTTWKGKTQ-TPEIIKRVVGLP-VDVEGMFGLAP-FL---RPPGL-------------
Nicotiana_tabacum[AIC85301.1]	SKGKVIL---DVT--------SNMAGLEVGGG---PWFNTWKGKAQ-MPEIVTRAINVP-VDLDGIFGLTP-FL---KPPGL-------------
Solanum_tuberosum[NP_001274927.1]	SKGEVIL---DVT--------SNMAGLEVGGG---PWFNTWEGKAE-MPEIVTRAINVP-VDLDGIFSCVPSLL---KPPGL-------------
Solanum_pennellii[ADZ24706.1]	SKGKVIL---DVT--------SNMAGLEVGGG---PWFNTWKGNAE-MPEIVTRAINVP-VDLDGIFSCVPSLL---KPPGL-------------
Helianthus_annuus[ABB52813.1]	ADGKLIL---DVT--------SNMAAVEVGGG---PWFNTWKGKTY-TPEVINRALNLP-VDVDGILGSFP-LL---KPPGL-------------
Lactuca_sativa[ADC91914.1]	ANGKLIL---DVT--------SDMAAVEVGGG---PWFNTWKGRTY-TPEIVSRAINLP-IDVEGILGSFP-LL---KPPGL-------------
Brassica_napus[AFB74209.1]	SKGKVIM---EAK--------SSMAAVEIGGG---PWFGTWKGDTSNTPELLKRSLQVP-LDIESVFGLVP-FF---KPPIL-------------
Arabidopsis_thaliana[NP_567906.1]	SKGKVIL---ETK--------SSMAAVEIGGG---PWFGTWKGDTSNTPELLKQALQVP-LDLESALGLVP-FF---KPPGL-------------
Theobroma_cacao[EOY29718.1]	TKGKLIL---DVK--------SDMAALEVGGG---PWFNTWKGETT-TPEVIKNALQVD-VDVEGIFGLAP-FF---KPPGL-------------
Gossypium_arboreum[KHG07989.1]	TKGKLIL---DVK--------SDMAALEVGGG---PWFNTWKGKTT-TPEVLKTALQVP-VDVEGIFGLAP-FF---KPPGLTHHQTSRRGS---
Gossypium_hirsutum[ABE41799.1]	TKGKVCY---SYLFDAKCNLRLQIIALGLGQT---IYFSSY--
Cucumis_sativus[KGN62825.1]	SKGKIVL---DVT--------SNMAALEVGGG---PWFNTWKGETT-TPEILKRALTTP-IDVDQAFNLLP-LF---KPPGL-------------
Eucalyptus_gunnii[AAP97931.1]	SKGKLIL---DVT--------SNMAAVEIGGG---PWFSTWKGKTS-TPELLSRALRVP-VDVDGFFGLAP-FL---KPPGL-------------
Hevea_brasiliensis[BAH10644.1]	SKGKIIL---DVT--------SDMAAVEVGGG---PWFNTWKGKTT-TPELLSRALRVP-LDVDGIFNFLP-LF---KPPGL-------------
Phaseolus_vulgaris[ACD50891.1]	SKGKIIL---NVS--------SNMAALEVGGG---PWFDTWKGKTS-TPAALRSALELP-IDVEAIFNLSS-VQ---TTWPLVNWFCCIPL----
Medicago_truncatula[KEH36047.1]	SKGKIIL---DVT--------SDMAALEVGGG---PWFNTWKGKTS-TPPVLSRAIGLP-VDVDGLYNLFP-LF---KPPGL-------------
Glycine_soja[KHN18183.1]	SKGKIIL---DVS--------SNMAALEVGGG---PWFNTWKGKTS-TPAALSRVLELP-IDVEGIFNPVP-LF---KPPGL-------------
Shuttleworthia_sp.[EUB12142.1]	WKWELVD---EIE--------AAHVGCEYGD--
Peptoclostridiu_difficile[EQJ63755.1]	-RHIKSWHFTQGG--------LERAGIDI--
Clostridium_sp.[EXG85387.1]	---ELIF---DLT--------SDHVSFEYVDR---
Clostridium_purinilyticum[KNF07861.1]	-INETIF---KGV--------GNCAGIEYGGEMQI---------------L--
Haloplasma_contractile[ERJ12496.1]	-THEIIY---SGV--------GKRAGIEYGGELRI---------------L--
Levilinea_saccharolytica[GAP18829.1]	--GQVLF---DEV--------GAHTGLEVMGDLPR---------------LLRG---
Flexilinea_flocculi[GAP40950.1]	--GKLLF---EGT--------GAHTGLEVMGNLPR---------------LLQK---
Clostridium_argentinense[KIE45238.1]	--YKKIF---DDI--------GTTVGLEISGDLED---------------LK---
Acholeplasma_oculi[CDR30325.1]	---QLVF---EDL--------GTDAGIEIMMKQA---
Mycobacterium_pseudoshottsii[GAQ37737.1]	-FGRVVF---DGT--------CGLAGLEVGSRPS---
Mycobacterium_kansasii[EUA18893.1]	-LGRVVF---DGT--------SELTGLEIGSRPPG--
Rhodococcus_rhodochrous[ETT23252.1]	-RGTLVW---ADE--------SPLAALEHGGLARA---RAELDRRG-APAD---ATDG------------PPLHGG-------------------
Corynebacterium_testudinoris[AKK08362.1]	-FGKHVW---TGT--------TTLAALEHGGLDRA---RAELRRRG-LDEG---LTHA------------PPRC---------------------
Armatimonadetes_bacterium[CUU10844.1]	------L---WQT--------SSACAAEWVEPSPDPRLGVWV---
