# Supplementary material for: Tocopherol Cyclases—Substrate Specificity and Phylogenetic Relations
Source: PLoS One. 2016 Jul 27;11(7):e0159629. doi: 10.1371/journal.pone.0159629 (PMC4963081; doi:10.1371/journal.pone.0159629)
Supplement: S2 Fig — The numbers following the species name, indicate the number of identical/similar residues between the sequences being compared. In the middle row, an aminoacid symbol indicates fully conserved residue, while '+' indicates a residue with strongly similar properties. The most conserved region in the sequence of photosynthetic organisms, shown in Fig 4, is highlighted in green. The analysis was performed using Blast algorithm. (PDF) [file pone.0159629.s002.pdf]

Shuttleworthia sp. 25/40

```
Query  272  GWIEWGGERFEFRDAPS--YSEKNWGGGFPRKWFWVQCN-----VFEGATGEVALTAGGG  324
            G + + G+ +   R   S  Y++KNWG  F R W W+  N     +        A   GGG
Sbjct  294  GRVVYNGQIYMVRPETSFGYADKNWGSDFTRPWIWLASNDLTSKISGDKLSNSAFAIGGG  353

Query  325  LRQLPGLTETYENAALVCVHYDGKMYEF  352
              ++  L    EN  L  + ++G+ Y+F
Sbjct  354  KPRIGNL--EVENGLLGAIWHEGEAYQF  379
```

Clostridium sp. 50/86

```
Query  91   FFEGWYFRVSIPEKRESFCFMYSVENPAFRQSLSPLEVALYGPRFTGVGAQILGANDKYL  150
            FFEGWYF+    +   +F   +S +    +                    QI+   + Y
Sbjct  21   FFEGWYFKHQNEDTVLAFIPGHSEDEKGVKHPF----------------LQIIWNENSYS  64

Query  151  CQYEQDSHNFWGDRHELVLGNTFSAVPGAKAPNKEVPPEEFNRRVSEGFQATPFWHQGHI  210
              +E+  +     R  ++LGN   ++ G K                   Q+     QG I
Sbjct  65   LDFEEKDYLVDRKRKRIILGNNIFSLTGVKVD----------------IQSKDISIQGMI  108

Query  211  CDDGRTDYAETVKSARWEYSTRPVYGWGDVGAKQKSTAGWPAAFPVFEPHWQICMAGGLS  270
                                      +G +   + +  G     P  E   +I
Sbjct  109  -------------------------RYGSLSPIEYTIMGPFQWIPFMECRHEIISMSHSL  143

Query  271  TGWIEWGGERFEFRDAPSYSEKNWGGGFPRKWFWVQCNVFEGATGEVALTAGGGLRQLPG  330
             G +   G+  +F     Y E + G  FPR + W+QCN F      +   A      +P
Sbjct  144  KGSLTVNGKVLDFDGEKGYIEGDRGRSFPRDYLWLQCNRFSQEASVMVSIA-----HIPF  198

Query  331  LTETYENAALVCVHYDGKMYEFVPWNGV  358
            +  +++    V + Y G+ Y    + GV
Sbjct  199  IGHSFQGCICV-IQYKGQEYRLATYLGV  225
```

Peptoclostridium difficile 19/34

```
Query  271  TGWIEWGGERFEFRDAPSYSEKNWGGGFPRKWFWVQCNVFEGATGEVALTAGGGLRQLPG  330
            +G I      F+   +  Y EK+ G  FP  + W Q N  E    + A      L  +P
Sbjct  144  SGTIRLNNRCFQIHGS-GYMEKDRGTSFPSAYAWFQSNNCE----DTASCFFFSLAHIPF  198

Query  331  LTETYENAALVCVHYDGKM  349
            LT +++    V + ++ ++
Sbjct  199  LTGSFQGCICVLMVHEKQL  217
```

Haloplasma contractile 74/138

```
Query  73   RELRTPHSGYHFDGTPRKFFEGWYFRVSIPEKRESFCFMYSVENPAFRQSLSPLEVALYG  132
            +E+R P   YH       +FEGWYF++       S+ F+     P   +     +   +
Sbjct  3    KEIRNPDL-YHGRRKKNNYFEGWYFKIVDVNNEHSYAFI-----PGISKGKELDDSHSF-  55

Query  133  PRFTGVGAQILGANDKYLCQYEQDSHNFWGDRHEL---VLGNTFSAVPGAKAPNKEVPPE  189
                    Q+L  N+      + D  +F  ++      +L N FS    + A
Sbjct  56   -------IQVLDGNNVTTEYIKFDQKDFHSEKKTFEIGILNNKFSLSKLSLAI-------  101

Query  190  EFNRRVSEGFQATPFWHQGHICDDGRTDYAETVKSARWEYSTRPVYGWGDVGAKQKSTAG  249
                                   +G T  + T++        + +  W D       + G
Sbjct  102  -----------------------EGETKLSGTLR-------FKNILKWPD-SILNPGSMG  130

Query  250  WPAAFPVFEPHWQICMAGGLSTGWIEWGGERFEFRDAPSYSEKNWGGGFPRKWFWVQCNV  309
            +   F   E + Q+C   G   G +    +  +F +   Y EKNWG  FP+ W W+Q N
Sbjct  131  YYNYFTFMECYSQVCALNGDIEGILTINNKEIDFTNGKVYIEKNWGKSFPQSWIWIQSNS  190

Query  310  FEGATGEVALTAGGGLRQLPGLTETYENAALVCVHYDGKMYEFVPWNGVVRWEMSPWGYW  369
            F      V+LT   G  ++P L     +  LV +  + ++Y+F   N
```

```
Sbjct  191  FNDRN--VSLTCSIG--RVPFLFGKTFSGFLVALSVNNQVYKFTTMNKSKMTINRSGNDV  246

Query  370  YITAENENHVVELEARTNEAGTPLRAPTTEVGLATACRDSCYGELKLQIWERLYDGSKGK  429
             I  + +N +++++ ++ +    ++    + G   +  D     LK ++  +L D +  +
Sbjct  247  RIDFKRKNLLLKIKTKS-KLNQFIQVKGPKNGEMVSVVDET---LKGEVVLKLIDLTTHE  302

Query  430  VILETKSSMAAVEIGG  445
            +I       A +E GG
Sbjct  303  IIYSGVGKRAGIEYGG  318
```

Clostridium purinilyticum 69/114

```
Query  73   RELRTPHSGYHFDGTPRKFFEGWYFRVSIPEKRESFCFMYSVENPAFRQSLSPLEVALYG  132
            +E+R P   YH     + FFEGWYF++        F F+     P   +  S
Sbjct  3    KEIRNPDL-YHGVNRKKNFFEGWYFKIVDKNCDNVFAFI-----PGIAKGNSL-------  49

Query  133  PRFTGVGAQIL-GANDKY-LCQYEQDSHNFWGDRHELVLGNTFSAVPGAKAPNKEVPPEE  190
             +F     Q+L G   KY   ++   S  F  D+  + + +   ++ G       +  E+
Sbjct  50   -KFNHSFIQVLDGKKVKYNYIKFNSSSFKFKKDKFSIAIEDNNFSLSGISL---NINNEK  105

Query  191  FNRRVSEGFQATPFWHQGHICDDGRTDYAETVKSARWEYSTRPVYGWGDVGAKQKSTAGW  250
             N   +  F+    W    +                                    + G+
Sbjct  106  DNISGNIKFENVKKWPDSIV--------------------------------NPGSMGF  132

Query  251  PAAFPVFEPHWQICMAGGLSTGWIEWGGERFEFRDAPSYSEKNWGGGFPRKWFWVQCNVF  310
                   E +  +C   G   G +   G+  +F     Y EKNWG  FP+ W W+Q N F
Sbjct  133  YNYLTFMECYSHVCCIDGNIIGGLNINGDYIDFTGGKVYIEKNWGRSFPKSWIWIQSNSF  192

Query  311  EGATGEVALTAGGGLRQLPGLTETYENAALVCVHYDGKMYEFVPWNGVVRWEMSPWGYWY  370
            +     VA T   G  ++P +  T+    LV +  + K Y+F   N   R +M
Sbjct  193  DNK--RVAFTCSIG--RVPFICTTFS-GFLVSIMVEDKFYKFTTIN---RSKMKI-----  239

Query  371  ITAENENHVVELEARTN  387
            I  +N+ ++V  + R N
Sbjct  240  IKKDNDVNIVFTKGRLN  256
```

Clostridium argentinense 44/87

```
Query  247  TAGWPAAFPVFEPHWQICMAGGLSTGWIEWGGERFEFRDAPSYSEKNWGGGFPRKWFWVQ  306
              GW +  P  E +  +        G +    E +       Y EK+WG  FP  W W+Q
Sbjct  132  IMGWYSFMPFMECYHGVVSINHSLKGTLIINEELYNLNSGKGYIEKDWGKSFPSSWIWLQ  191

Query  307  CNVFEGATGEVALTAGGGLRQLPGLTETYENAALVCVHYDGKMYEFVPWNGVVRWEMSPW  366
            CN F+ +     L+    + ++P L   +    L  ++ DG +  F  + G +  +++
Sbjct  192  CNNFKNSNSSFMLS----IAKIPWLKNHFV-GFLGFLYIDGILLNFATYTGAIIKDITVI  246

Query  367  -GYWYITAENENHVVELEARTNEAGTPLRAPTTEVGLATACRDSCYGELKLQIWERLYDG  425
                +IT E++N  ++L     + G  L+AP++    +    ++S   ++ +Q++ + Y
Sbjct  247  KNKLFITIEDKNFKIKL-CSIKKDGVELKAPSSG-KMERTIKESLNSKIHIQVFNKQY--  302

Query  426  SKGKVILETKSSMAAVEIGG  445
               K I +   +   +EI G
Sbjct  303  ---KKIFDDIGTTVGLEISG  319
```

Acholeplasma oculi 41/83

```
Query  260  HWQICMAGGLS-----TGWIEWGGERFEFRDAPSYSEKNWGGGFPRKWFWVQCNVFEGAT  314
            H+  C  G LS     TG I+     +      +Y EK+WG  FP+ + W+Q N F+
Sbjct  138  HFMECYHGILSMDSKVTGSIQINKTTYNIDQEKAYIEKDWGKSFPKGYVWLQSNHFKNKN  197

Query  315  GEVALTAGGGLRQLPGLTETYENAALVCVHYDGKMYEFVPWNG--VVRWEMSPWGYWYIT  372
                 +       +P +  T++   +  +H +G  Y F  +N   V +  +      +I
```

```
Sbjct  198  TSFMFSYA----YIPFVFFTFKGLIINLIH-EGIEYRFATYNNSKVKKMVIENNKVLFII  252

Query  373  AENENHVVELEARTNEAGTPLRAPTTEVGLATACRDSCYGELKLQIWERLYDGSKGKVIL  432
             +  +  +E+ A T      L +P+  + + T  ++   GE+ +++++      K +++
Sbjct  253  KKG-SLTLEVSA-TKSDDIELVSPSMGMMIHT-IKEGLSGEITVKLYK------KNQLVF  303

Query  433  ETKSSMAAVEI  443
            E   + A +EI
Sbjct  304  EDLGTDAGIEI  314
```

Levilinea saccharolytica 45/156

```
Query  84   FDGTPRK--FFEGWYFRVSIPEKRESFCFMYSV----ENPAFRQSLS  124
            + G  RK  FFEGWY+++   ++R     +  V    +  AF Q L
Sbjct  12   YHGFKRKPPFFEGWYYKLVSADERHKVAVIPGVILGRDEHAFVQVLD  58
Query  249  GWPAAFPVFEPHWQICMAGGLSTGWIEWGGERFEFRDAPSYSEKNWGGGFPRKWFWVQCN  308
            GW A  P  E +  +        G +   G+  +F     Y EK+WG  FP  W W Q N
Sbjct  129  GWYAWVPRMECYHGVLSFSHSLQGTLTLNGKVMDFSGGRGYIEKDWGQSFPAAWIWCQSN  188

Query  309  VFEGATGEVALTAGGGLRQLPGLTETYENAALVCVHYDGKMYEFVPWNG  357
             F  A     LTA   +  +P +   +    +V +  DG+++ F  ++G
Sbjct  189  HF--ANPSACLTASVAV--IPWVGRAFR-GFIVGLMLDGQLHRFATYSG  232
```

Flexilinea floccule 51/85

```
Query  249  GWPAAFPVFEPHWQICMAGGLSTGWIEWGGERFEFRDAPSYSEKNWGGGFPRKWFWVQCN  308
            GW A FP  E +  I        G + W G   +F     Y EK+WG  FP  W W+Q N
Sbjct  129  GWFAWFPHMECYHGILSFSHTLQGSLTWNGTEMDFSGGRGYIEKDWGKSFPSAWIWLQSN  188

Query  309  VFEGATGEVALTAGGGLRQLPGLTETYENAALVCVHYDGKMYEFVPWNG--VVRWEMSPW  366
             F  A  +  +TA   +    G         +V    DG ++ F  +NG  ++  ++
Sbjct  189  HF--AATDACITASVAVIPWTGFD---FRGFIVGFWLDGSLHRFATYNGSRIISLQIFD-  242

Query  367  GYWYITAENENHVVELEARTNEAGTPLRAPTTEVGLATACRDSCYGELKLQIWERLYDGS  426
             Y     +N    + L+A   + G  L     ++G       +   E++L+  +
Sbjct  243  DYVSWVLQNRQFRLSLKAFRVQGGLLLGPTRQDMGQRVMETLNATVEVRLETLQ------  296

Query  427  KGKVILETKSSMAAVEIGGGPWFGTWKGDTSNTPELLKQ  465
             GK++ E   +   +E+ G            N P LL++
Sbjct  297  -GKLLFEGTGAHTGLEVMG-----------NLPRLLQK  322
```

Mycobacterium pseudoshottsii 53/92

```
Query  252  AAFPVFEPHWQICMAGGLSTGWIEWGGERFEFRDAPSYSEKNWGGGFPRKWFWVQCNVFE  311
            ++ P    +W      GG ++G + + GE + F  A  Y E+NWG GFP  W+W Q + F+
Sbjct  140  SSIPFLNQYWHPYRLGGNASGTVGFNGETWSFDSARLYCERNWGTGFPEHWWWGQAHDFD  199

Query  312  GATGEVALTAGGGLRQLPGLTETYENAALVCVHYDGKMYEFVPWNGVVRWEMSPWGYWYI  371
             A   VA +  GGL Q   +    ++   V V    ++    P   VVR +++  G+W +
Sbjct  200  NADASVAFS--GGLLQFGPIR---QDVTGVVVRLGDEVIRVTP-PAVVRSQITD-GHWRV  252

Query  372  TAENENHVVELEARTNEAG---TPLRAPTTEVGLATACRDSCYGELKLQIWERLYDGSKG  428
             A + ++ +ELE     +G    P+  P     + T   D  +   +L    R +    G
Sbjct  253  RAFSLHYQIELEGEGAGSGPHTLPVPVPAQRHNVDT---DFEHLAGRLHCVVRKF----G  305

Query  429  KVILETKSSMAAVEIGGGP  447
            +V+ +    +A +E+G  P
Sbjct  306  RVVFDGTCGLAGLEVGSRP  324
```

Mycobacterium kansasii 60/225

```
Query  252  AAFPVFEPHWQICMAGGLSTGWIEWGGERFEFRDAPSYSEKNWGGGFPRKWFWVQCNVFE  311
            ++ P    +W     GG ++G +E+    + F DA  Y E+NWG GFP +W+W Q + F+
Sbjct  148  SSIPFLNQYWHPYRLGGKASGTVEFSDSTWTFDDARLYGERNWGAGFPERWWWGQAHDFD  207

Query  312  GATGEVALTAGGGLRQLPGLTETYENAALVCVHYDGKMYEFVPWNGVVRWEMSPWGYWYI  371
            GA   VA +  GGL QL  +    ++   V V    ++    P   +VR ++   G+W I
Sbjct  208  GADVSVAFS--GGLLQLGPIR---QDVTGVVVRLGDEVIRMTP-PALVRSQVGD-GHWRI  260

Query  372  TAENENHVVELEARTNEAG-----TPLRAPTTEVGLATACRDSCYGELKLQIWERLYDGS  426
             A    + + L+   ++AG      P+  P     + T   +   G L   + +
Sbjct  261  RAFTPLYQIWLDG--DDAGRDPHVLPVPLPAQRRNVDTDF-EHLAGRLHCVVRK------  311

Query  427  KGKVILETKSSMAAVEIGGGP  447
             G+V+ +  S +  +EIG  P
Sbjct  312  LGRVVFDGTSELTGLEIGSRP  332
Query  92   FEGWYFRVSIPEKRESFCFMYSVE  115
             EGW++R++          + SV
Sbjct  41   MEGWFWRLTDSASDRVVVGLCSVN  64
```

Corynebacterium testudinoris 37/47

```
Query  254  FPVFEPHWQICMAGGLSTGWIEWGGERFEFRDAPSYSEKNWGG-GFPRKWFWVQCNVFEG  312
             P    +W   +  G ++G    GGE +EF  A  YSEKNWG  GFP  W+W Q   F
Sbjct  162  IPALNQYWHPWLPSGRASGTARVGGETWEFDGAQVYSEKNWGREGFPESWWWGQAQGFAE  221

Query  313  ATGEVALTAGGGLRQLPGLTETYENAALVCVHYDGKMYEF  352
                VA  AGG +   P    T E  ALV +  DG++
Sbjct  222  PGASVAF-AGGVVTSGP---LTVEVTALVVMLPDGRVIRL  257
```

Rhodococcus rhodochrous 91/350

```
Query  92   FEGWYFRVSIPEKRESFCFMYSVENPAFRQSLSPLEVALYGPRFTGVGAQILG-ANDKYL  150
             EG+++R + P        +  V N A   S + L +A +   F    A   G A+ + L
Sbjct  27   MEGYFWRFTQPGTGRVVIALCGV-NRAADGSWATLGLAAHPGGFLRTTAHPEGFADPRRL  85

Query  151  CQYEQDSHNFWGDRHELVLGNTFSAVPGAKAPNKEVPPEEFNRRVSEG---FQATP----  203
                  +     DR  + LG+       A+   +   P  + RR   G   F A P
Sbjct  86   GASAGTAFRGEPDRLRVDLGDD------ARLDVRVTAPRPWPRRRFGGSSVFHAVPALNQ  139

Query  204  FWHQGHICDDGRTDYAETVKSARWEYSTRPVY  235
            +WH   +  DGR +    +    W      VY
Sbjct  140  YWHPWLL--DGRAEGEAVLGGETWNLDGAQVY  169
Query  253  AFPVFEPHWQICMAGGLSTGWIEWGGERFEFRDAPSYSEKNWG-GGFPRKWFWVQCNVFE  311
            A P    +W   +  G + G    GGE +    A  Y+EKNWG GGFP  W+W Q + F
Sbjct  133  AVPALNQYWHPWLLDGRAEGEAVLGGETWNLDGAQVYAEKNWGRGGFPESWWWGQAHGFT  192

Query  312  GATGEVALTAGGGLRQLPGLTETYENAALVCVHYDGKMYEF-VPWNGVVRWEMSPWGYWY  370
             +   VA  AGG +   P  TE     A+V    DG +     P    VR  ++    W
Sbjct  193  DSAACVAF-AGGQVHAGPLRTEV---TAVVVALPDGPVIRLGNPVVSPVRAHVAD-ERWS  247

Query  371  ITAENENHVVELEARTNEAGT---PLRAPTTEVGLATACRDSCYGELKLQIWERLYDGSK  427
            +        VE+EA    +     P+  P     +  A  +   G L + + +R
Sbjct  248  LFGRGPRWTVEIEATAPLSAAHVLPVPRPLERRNVPGAL-EHLAGSLSVTVRDR------  300

Query  428  GKVILETKSSMAAVEIGG  445
            G ++   +S +AA+E GG
Sbjct  301  GTLVWADESPLAALEHGG  318
```

Armatimonadetes bacterium 57/81

```
Query  253  AFP---VFEPHWQICMAGGLSTGWIEWGGERFEFRDAPSYSEKNWGGGFPRKWFWVQCNV  309
            AFP      P+W + ++G      IE  GER      P+     WG G+  +W W  CN
Sbjct  139  AFPRTKYVAPNWNVRLSGV-----IEHEGERVSLERVPAQQAHLWGTGYAERWVWAHCNA  193

Query  310  FEGATGEVALTAGGGLR----QLPGLTETYENAALVCVHYDGKMYEFVPWNGVVRWEMSP  365
            FEG+   V       +R     +LP L        ++C++  G  Y  VP     R   S
Sbjct  194  FEGSPDVVFEGLEAQVRLGRFRLPPLR-------MLCLYAGGHWY--VPRPSWGRDSQSA  244

Query  366  WGYWYITAENENHVVE---LEARTNEAGTPLRAPTTEVGLATACRDSCYGELKLQIWERL  422
             G+W     +    +E     A     G   R P+ E      C +S    L+L     RL
Sbjct  245  IGWWRFRGASRGFTLEGTVQAAVERMLGARYRTPSGE---ERWCHNSKLASLQL----RL  297

Query  423  YDGSKGKVILETKSSMAA--VEIGGGPWFGTW  452
                  + + +T S+ AA  VE    P  G W
Sbjct  298  RGSGGSETLWQTSSACAAEWVEPSPDPRLGVW  329
```
